# Beyond antimalarial stock-outs: implications of health provider compliance on out-of-pocket expenditure during care-seeking for fever in South East Tanzania

**DOI:** 10.1186/1472-6963-13-444

**Published:** 2013-10-27

**Authors:** Inez Mikkelsen-Lopez, Fabrizio Tediosi, Gumi Abdallah, Mustafa Njozi, Baraka Amuri, Rashid Khatib, Fatuma Manzi, Don de Savigny

**Affiliations:** 1 Swiss Tropical and Public Health Institute, Basel, Switzerland; 2 University of Basel, Basel, Switzerland; 3 Centre for Research on Health and Social Care Management (CERGAS), Bocconi University, Milan, Italy; 4 The Ifakara Health Institute, Ifakara, Tanzania

**Keywords:** Health worker behaviour, Informal charges, Stock-outs, Antimalarial, Tanzania

## Abstract

**Background:**

To better understand how stock-outs of the first line antimalarial, Artemisinin-based Combination Therapy (ACT) and other non-compliant health worker behaviour, influence household expenditures during care-seeking for fever in the Ulanga District in Tanzania.

**Methods:**

We combined weekly ACT stock data for the period 2009-2011 from six health facilities in the Ulanga District in Tanzania, together with household data from 333 respondents on the cost of fever care-seeking in Ulanga during the same time period to establish how health seeking behaviour and expenditure might vary depending on ACT availability in their nearest health facility.

**Results:**

Irrespective of ACT stock-outs, more than half (58%) of respondents sought initial care in the public sector, the remainder seeking care in the private sector where expenditure was higher by 19%. Over half (54%) of respondents who went to the public sector reported incidences of non-compliant behaviour by the attending health worker (e.g. charging those who were eligible for free service or referring patients to the private sector despite ACT stock), which increased household expenditure per fever episode from USD0.14 to USD1.76. ACT stock-outs were considered to be the result of non-compliant behaviour of others in the health system and increased household expenditure by 21%; however we lacked sufficient statistical power to confirm this finding.

**Conclusion:**

System design and governance challenges in the Tanzanian health system have resulted in numerous ACT stock-outs and frequent non-compliant public sector health worker behaviour, both of which increase out-of-pocket health expenditure. Interventions are urgently needed to ensure a stable supply of ACT in the public sector and increase health worker accountability.

## Background

### Universal Coverage

The key goals of universal coverage as outlined in the *World Health Report* 2011 require knowledge about: the proportion of the population covered by health services, together with widening the range of available services and reducing the proportion of total costs born by individuals [1]. In order to meet these objectives, some countries have focused their efforts on achieving universal coverage for selected health topics by developing essential medicines lists, which, according to the WHO, identify medicines that “satisfy the priority health care needs of the population” and should be available at prices the public can afford, with assured quality.

### Essential Medicines in Tanzania

Tanzania’s current 2007 National Essential Medicines List includes medicines for 26 priority conditions, one of which is malaria [2]. Malaria is a leading public health concern in Tanzania especially for children under five years and pregnant women [3]. Expenditure on malaria programs make up 19.4% of total health expenditure and 1.6% of GDP [4]. The disease places a large burden on the health sector accounting for around 40% of out-patient department visits in 2008 [5]. Since 2006, the first-line antimalarial for uncomplicated malaria in Tanzania is an Artemisin-based Combination Therapy (ACT) - Artemether lumefantrine (Alu). However, given the high costs of ACT, Tanzania was granted USD75 million from the Global Fund to fight AIDS, Tuberculosis and Malaria (henceforth referred to as the ‘Global Fund’) during its Round 4 in 2005 (and currently through Round 9) to purchase ACTs (which come in four doses based on patient weight). Tanzania’s National Malaria Control Programme is responsible for forecasting ACT demand and managing Global Fund grants for malaria [6], while the Pharmaceutical Supply Unit is charged with setting policy on medicines and supplies and budgeting for medicines. It also monitors the use of funds and supervises health facilities. The Medical Stores Department (MSD) handles ACT procurement, storage and distribution together with other medicines [7]. Health facilities order ACTs along with other medicines through a ‘pull’ system via the Integrated Logistic System (ILS) [8]. The ACTs are provided at no charge to health facilities and, according to policy, are dispensed free to children under the age of five and to adults over 60 years of age [9]. Those covered by a health insurance fund (National Health Insurance Fund or the Community Health Fund) (http://www.nhif.or.tz/) are also exempt from payment at the health facility [10,11]. Others pay a user fee of TZS1000 (USD0.70) (2007 fee) [12], although independent confirmation has found this to vary and can be as low as TZS500 per visit. According to the Integrated Management of Childhood Illnesses Guidelines (IMCI), ACTs are given as a presumptive treatment in the absence of diagnostic tests when a child reports with fever without other symptoms such as rapid breathing or runny nose which could indicate pneumonia or a common cold [13]. Apart from ACT, other antimalarials are Sulphadoxine – Pyrimethamine (SP), which is recommended only as intermittent preventive treatment during pregnancy, and quinine which is a second-line treatment administered to pregnant women in their first trimester [14].

The design of the ACT procurement and delivery system should ensure availability in the public sector. However, Tanzania along with its neighbours, has been experiencing public sector ACT stock-outs over the past five years [6,15-21] which cripples the delivery of health care services. Our objective is to investigate the impact of public sector ACT stock-outs together with health worker behaviour on care-seeking during a fever episode and the associated household expenditures.

## Methods

Data for our case-study were obtained from the Ulanga District in South East Tanzania with an estimated population of 265,203 according to the 2012 census and with a malaria parasite prevalence rate in children under five years of age as tested by a Rapid Diagnostic Test (RDT) of 13.0% in 2011 [22]. Health service infrastructure in Ulanga is composed of two hospitals (one public), three health centres and 30 dispensaries (16 public). The estimated population per facility is 4,571. We combined data from three surveys carried out in Ulanga District between November 2009 and August 2011.

### Household behaviour and expenditure

We used longitudinal survey data on household costs drawing on the International Network for the Demographic Evaluation of Populations and Their Health (INDEPTH) Effectiveness and Safety Studies of Anti-malarial Drugs in Africa (INESS) methodology, starting in September 2009 until present [23]. Rolling daily household surveys in the Ifakara Health and Demographic Surveillance Site (HDSS) identify fever episodes using a two week recall where a randomly pre-selected group is chosen for an in-depth questionnaire on their behaviour and expenditures. There was no clinical verification that a fever episode was a case of malaria. Data on individual treatment seeking pathways, access to treatment, outcomes, services at provider level and household costs are captured, together with other key indicators such as the different treatments and sources of medicines as well as the total cost. Direct financial costs are considered to include direct medical costs such as consultation fees, prescription fees and charges for medicines, together with non-medical costs such as transport, accommodation, food, water and mobile phone use in addition to any gift payments. Respondents are grouped into public or private sector based on their first provider, even if they received medicines from the other.

### Household demographics

As part of the Ifakara HDSS, households are visited three times a year and once a year an asset survey is administered. The socio-economic status of households is based on 15 dichotomous variables using principle component analysis (PCA) [24,25]. The index was constructed for 5,676 of the households from the following dichotomous variables: ownership of a bicycle (65% of households); radio (69%); mobile phones (43%); watch (6%) and iron (5%); living in rented accommodation (11%); as well as various characteristic of the dwelling such as: mud floor (83%); cement floor (9%); stone walls (33%); brick walls (6%); grass roof (9%); tin roof (1%); kerosene fuel (20%); electricity (2%) and type of sanitary facilities including presence of toilet (94%). The first principle component explained 23% of the variability in socio-economic scores. Greatest weight was given to having a cement floor (0.38), the use of kerosene for cooking fuel (0.34) and ownership of a mobile phone (0.30). Households were classified into wealth quintiles based on their PCA sores and assigned their own socio-economic scores index. Household and health facility Geographic Information Systems (GIS) coordinates were also collected in the HDSS. Health facility catchment areas were determined using the ArcGIS software [26] to create 1km Euclidian buffers around health facilities.

### SMS for life

We used information provided by the *SMS for Life* project [27] which monitors weekly stock-levels via a mobile phone Short Messaging System (SMS) on all four dose levels of the ACT *Coartem* in 35 health facilities in Ulanga, of which eight were also covered by the Ifakara HDSS. Of these eight, we excluded two as they were in the same village and therefore could not be associated with individual catchment households resulting in a total of six health facilities included (five dispensaries and one health centre). We identified the number and pattern of weeks where they were stocked out of all four doses of ACT. ACT stock-out was defined when all four ACT doses were stocked out since health workers cope with a stock-out of one dose by dividing blister packets from others. If in a given week the health worker did not respond to the SMS, this was considered a stock-out only if it was a preceded or followed by a reported stock-out. From (and including) November 2009 until (and including) August 2011, there were 576 health facility weeks of data of which 82 (14%) reported total stock-out.

Informed consent was obtained for this study from the Tanzania National Institute for Medical Research (NIMR/HQ/R.8a/Vol.IX/998).

Univariate logistic models were fitted to assess the effect of predictors on household expenditure during care seeking for fever and whether these varied across wealth quintiles. All analysis was done using Stata 10.

We defined non-compliance for user fee charges in the public sector as charging patients user fees contrary to policy (i.e. for patients who were under the age of five or over the age of 60 or who were covered by health insurance) or when individuals who did not qualify for one of the above exemptions were charged over TZS1,000 (using the higher user fee). Non-compliance for medicines management was defined as cases where a fee of more than TZS1,000 was charged in the public sector for medicines as ACTs were supposed to be dispensed for TZS300 (or free), and a combination of antipyretic and other basic medicines would cost no more than TZS1,000. Non-compliance for medicines management was also defined in cases when a monotherapy (SP or quinine) was dispensed, either in the public or private sector, apart from when this was given for free in a public sector facility to a female of reproductive age, according to Standard Treatment Guidelines for women in their first trimester of pregnancy who should receive quinine for the treatment of uncomplicated malaria [2]. Referral to the private sector to purchase medicines during stock-in periods was also considered non-compliant behaviour by health workers. Fever cases not receiving an antimalarial were not considered as non-compliant as there may have been other symptoms that suggested another diagnosis, or a negative test from a RDT. We believe that ACT stock-outs in the public sector are in part a result of the inability of healthcare workers to reliably ensure that medicines are ordered appropriately and on time (in addition to non-compliant behaviour of individuals higher up on the supply chain) and therefore incidents of care seeking during an ACT stock-out were also attributed to non-compliant behaviour of health workers. Furthermore, we believe that it is reasonable to assume that the cost of non-compliant behaviour in the public sector resulting in stock-outs also results in costs experienced by households seeking care (purchasing ACTs) in the private sector if these occurred during an ACT stock-out. This is because only 9% of total care seeking chose the private sector during times when the public sector was stocked in, as well as the fact that news of public sector ACT stock-outs spreads quickly in times of frequent stock-outs of commonly sought products. Some patients may therefore go directly to nearby private sector facilities for treatment. While these costs may not have arisen directly as a consequence of non-compliant health worker behaviour, they nonetheless would not have arisen if health staff and others in the procurement and supply chain system had been ‘compliant’ with their own policies and processes to avoid stock-outs. Finally, the cost to the household of non-compliance may be defined as the difference between the average direct cost for compliant behaviour and the average direct cost for the non-compliant behaviour.

## Results

### General characteristics

There were 439 INESS fever interviews in the Ulanga District between November 2009 and August 2011. From these, we excluded respondents whose closest health facility was not one of the six, or who sought treatment from another source such as from a friend or family, or a traditional healer. We also excluded respondents who went to a hospital as this involved cases of complicated malaria requiring inpatient care. Two outliers were removed as they reported excessively high costs (TZS12,000 on medicines). Of the remaining 333 fever cases, 192 (58%) sought initial care from the public sector and 141 (42%) from the private sector. The most common private sector outlets were Accredited Drug Dispensing Outlets (ADDOs) (62%); others went to private pharmacies and shops.

**Figure 1:**
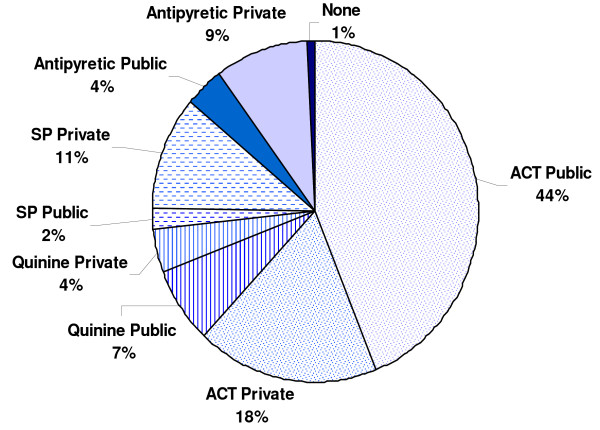
Distribution of medicines taken for fever, by type and source, Ulanga District, Tanzania, November 2009 – August 2011.

86% of fever cases received an antimalarial (ACT, quinine or SP) (see Figure 1) of which the most common was ACT (72%). The rest took an antipyretic such as aspirin, panadol, ibuprofen or diclofenac.

ACT and quinine were most commonly obtained from the public sector, whilst SP was predominantly obtained in the private sector. The majority (58%) of those who received SP were men, against policy.

**Figure 2:**
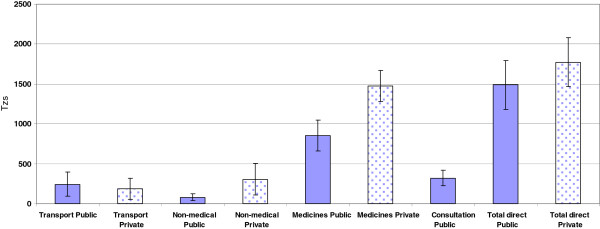
**Average cost (TZS) of fever treatment in both the public and private sector, Ulanga, Tanzania, November 2009 – August 2011.** Note: Non-medical costs include: food, water, lodging, phone, gift payments. TZS 1,434 = USD1 (average exchange rate during the study period 2009- 2011). Source: Bank of Tanzania. There were no consultation costs in the private sector.

The costs experienced by respondents seeking treatment in each of the two sectors (private and public) are illustrated in Figure 2.

The total direct cost of seeking treatment for fever was TZS285 (19%) higher in the private than in the public sector (95% CI: 139 – 857). This was largely due to higher (73%) average medical costs where the difference between both means was TZS736 (95% CI: 428 – 1044). Non medical costs where also nearly four times higher (95% CI: 124 – 448) in the private sector compared to the public sector, although transport costs were lower. In addition, patients seeking care in the private sector were not charged a consultation fee, which made-up about one quarter of the direct public sector treatment.

**Figure 3:**
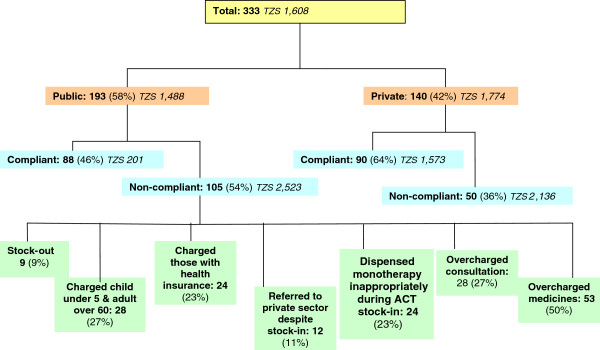
**Care seeking and average direct costs during fever episodes, Ulanga District, Tanzania, November 2009 – August 2011.** Note that the number of non-compliant cases (i.e. cases subject to non-compliant behaviour of health care workers) in the public sector does not sum to total non-compliant cases due to some individuals experiencing more than one non-compliant behaviour at the health facility during fever treatment.

A more detailed breakdown of these costs, showing cases which were subjected to non-compliant behaviour of any sort by health care workers can be seen from Figure 3. Non-compliant behaviour was relatively more common in the public sector, with overcharging for medicines being the most common form.

### Cost of non-compliant behaviour

The cost of non-compliant health worker and health system behaviour in the Ulanga District for those seeking fever treatment in the period 2009-2011 is estimated as USD1.62 or TZS2,322 per episode of fever. When health workers and shop workers are compliant, the cost is lower in the public sector than the private sector (USD0.96, TZS1,372). However, in non-compliant cases, the reverse is true with the cost of care being marginally lower in the private sector compared to the public sector (USD0.27, TZS387).

### Stock-out analysis

Out of the total sample, 7% of respondents sought treatment for fever during an ACT stock-out and their direct costs were 21% higher at TZS1,921 (95% CI: 1251-2590) compared to those who sought treatment during a stock-in [TZS1,584 (95% CI: 1353-1815)]. The majority of these patients (79%) received their medicines from the private sector which explains the high direct costs. Of these, 53% obtained an ACT. Due to the small sample size of respondents seeking care during an ACT stock-out, we were unable to confirm whether the increase in costs during fever seeking was statistically significant (p = 0.44).

PCA scores were available for 286 households and regression analysis suggested that those in the higher socio-economic quintile tended to use the private sector more, although the difference was not statistically significant. The consumption of antimalarials did not vary significantly across socio-economic quintiles, although the poorest were more likely to take a (cheaper) antipyretic. Healthcare seeking expenses did not vary significantly across socio-economic quintiles, although those in the higher socio-economic quintile tended to spend more (TZS126, 95% CI: -14.78 – 267.14, p = 0.08).

### Limitations

The INESS data included hospitalization costs which were a lump sum of medical costs, laboratory costs and bed costs. As some respondents who went to a dispensary reported only hospitalization costs, we distributed these costs between medicines (80%) and consultation (20%) on the basis of the observed average distribution among respondents who incurred both costs. There were also inconsistencies in the household data where for example, people going to a private shop reported having bed costs, or reporting no cost. Also, it is likely that the two week recall of respondents would have affected data quality. Finally, *SMS for Life* includes long periods of non-reporting; for example, in 2011 more than one in three weeks (37%) had missing data. Furthermore, *SMS for Life* only counts boxes in the store room, not in the dispensing room, therefore ACT stock-out rates could be inflated.

## Discussion

The frequent ACT stock-outs which have occurred in Tanzania have not been well documented in the literature. The aim of this case-study was to better understand household behaviour and expenditures during care-seeking for fever in the Ulanga District and whether they varied during an ACT stock-out. However, from our results stock-outs accounted for a relatively small proportion (less than 10%) of the non-compliant behaviour in the system experienced by respondents when attending the public health facilities for treatment. By far, the largest contributor to non-compliant behaviour was overcharging for medicines and/or consultations and not respecting health insurance status, as also noted in Tanzania by others [28-30]. Non-compliant health worker behaviour has also been found in other African countries such as Uganda [20,31] Ethiopia [32] and Zambia [19].

Overcharging for services or goods that are supposed to be free could be an outcome of various dysfunctional elements across the health system. Within the information system, overcharging could be a result of lack of information among patients who may be under informed on their rights and entitlements. Indeed, during our field visits, we did not observe any posters displayed at health facilities stating the fee-for-service or patient rights. Similarly, lack of information could be occurring on the supply side where health facility staff are unaware of the fee-for-service guidelines. Health system stewards could consider strengthening information flows by designing interventions aimed at increasing health worker training on user fee policies followed by reminder messages using mobile phones.

Overcharging could also be occurring due to weaknesses in the financing system especially with regards to payment of salaries to health workers, who may be overcharging as a coping mechanism, either because of salary delays or because funding to the health facility is irregular and limited. If this is the case, then stewards would need to review the remuneration system for health workers and streamline the flow of funds to health facilities. Another potential intervention could be to allow more access to funds received at the health facilities through cost sharing schemes such as insurance funds which could then be used to purchase medicines from other sources when needed. This would reduce the frequency of stock-outs and informal charges, empower the health workers, and build trust in the health care system among the community.

Overcharging for health care services results in a dual burden to the health system, both by potentially discouraging patients from receiving care, and possibly impoverishing them when they do seek care. Considering that the commodity supply cost of ACTs together with other technologies used to prevent malaria such as bed nets and RDTs are nearly fully donor funded in Tanzania, it is perhaps surprising that the 2009/2010 National Health Accounts found that approximately 40% of total malaria expenditure came from households [4]. This is particularly concerning as out-of-pocket costs for medicines tend to be the second largest family expenditure item after food in developing countries [33], resulting in equity and impoverishment implications, together with creating barriers for universal coverage. Socio-economic considerations are important as our results indicated that the least poor tended to use the private sector more than the poorest households. There could be a combination of demand and supply factors that contribute to this observation, on the demand side, the least poor may want to avoid long queues and therefore go directly to the private sector to purchase medicines. On the supply side, unpublished work from the Ifakara HDSS running in this district has geo-positioned all households and has socio-economic status of a large sample of households, it shows that the least poor quintile is more commonly located in the centre of large villages (merchants, teachers, other elites, etc) while the poorest are more scattered and distributed in and between small villages (peasant farmers, etc.).^a^ The implication of this is that the poor rely on the public health care system to provide health services, and when these are either not available, or not accessible, it becomes an indicator of health system failure.

At just under 10% of the non-compliant behaviour observed in our study, ACT stock-outs are a serious issue for those experiencing them, especially when they concern essential medicines for a leading cause of a life threatening condition. Stock-outs are a result of a combination of governance challenges and weak system design. The design of the current system is such that Tanzania relies entirely on donor support for its ACTs, most of which comes from the Global Fund and President’s Malarial Initiative (PMI) [34]. Relying on donor support puts Tanzania at risk of having to abide by external bureaucratic requirements. For example, a report by the Local Fund Agent for the Global Fund in 2009 reported stock management issues which resulted in the Global Fund withholding around USD 1.2 million for the purchase of ACTs [35]. A delay in Round 7 application was a consequence of having to restructure the grant to host the innovative financing mechanism ‘Affordable Medicines Facility for malaria’ (AMFm) which aimed to lower the price of ACTs [36]. This resulted in Tanzania only submitting their proposal in June 2009 and signing the AMFm a year later [37,38].

Another health system design feature which would have contributed to the ACT stock-out was the nature of the supply chain system which is based on a ‘pull’ system. That is, medicines availability is entirely based on whether health facilities order the correct amount of medicines at the right time from MSD.

Tanzania could consider redesigning the medicine supply system to simplify the way that funds are transferred to the MSD for procurement. Understanding the resource constraints of health budgets in developing countries, Tanzania could take the opportunity of subsidised ACTs through the AMFm program (first orders were received July 2011) to allocate a small portion of domestic funds to co-finance ACT purchases, including creating a buffer stock which would mitigate future stock-outs. With the introduction of RDTs to accurately diagnose malaria, fewer ACT doses will be required. Health system stewards could also consider carrying out an external assessment of the medicines quantification, procurement and supply system to identify and address barriers, potentially considering moving towards a system which did not rely entirely on health workers placing medicines orders in on time.

There are also a range of governance challenges centred around lack of accountability and transparency which can contribute to stock-outs. Tools like *SMS for Life* can greatly increase transparency of medicines stocks and identify where the barriers are, but this is only if they are being widely accessed, which at present it is not due to the website being password protected. Therefore, many involved in the forecasting, procurement and delivery of ACTs may be unaware of stock-outs at the dispensary level as they only monitor stock levels in the national or zonal warehouse. If there was more transparency, perhaps accountability would increase along the supply chain and which may reduce any unjustifiable referral to the private sector, especially when ACTs are in stock.

Our results indicate that in a stock-out period household expenditure during care seeking for fever increased (this was not statistically significant). However, average district ACT stock-out rates are increasing. For 2009, 2010, and 2011, district ACT stock-out rates were, respectively, 8.3%, 11.4% and 34.1%, and so a district analysis today may yield statistically significant increases in household expenditure results. The predominant periods of ACT stock-outs were November 2009; July to August 2010; and the most severe was between March – August 2011, corresponding to the peak period of malaria transmission. Thus, in addition to the economic consequences of ACT stock-outs, it would not be unreasonable to expect sever health consequences in affected communities, although this remains to be established.

The irregular public sector ACT availability could have been one of the principal factors contributing to nearly half the respondents going to the private sector for fever treatment, especially since cost of care in the private sector for fever treatment was only slightly higher (19%) than in the public sector. Our case-study does not examine the private sector ACT stock, which may have been higher following the earlier introduction of AMFm funded ACTs compared to the public sector. Another factor dissuading patients from seeking care at public health facilities was the prevalent practice of overcharging. Overcharging (an average, per case, of TZS200 per patient) was also reported in a GIZ 2011 study on the availability of medicines which found in one district that health facilities were overcharging patients to pay for security services [18].

Our overall costing results differ from those of Somi *et al.* (2007) who calculated the direct cost of fever/malaria treatment in the same Ifakara HDSS site in 2004 and found the public sector to be 61% more expensive that the private sector [39]. Using GDP deflators from the World Bank, the average direct cost in 2004 was TZS1,184, 50% or so higher than the average cost during our study (TZS767). A direct comparison is difficult because in 2004 the first line antimalarial was SP which was at the time much more inexpensive than ACT. Choloroquine was also still popular in the shops and very inexpensive.

The annual number of malaria cases reported in public health facilities in Tanzania is estimated at between 14 – 18 million [40]. If over half of these cases experienced additional costs (USD1.62) due to the non-compliant behaviour of health system employees, the estimated total additional expenditure born by households would be between USD12.2 - USD15.7 million. This represents about 3.6 - 4.6% of the total health expenditure on malaria in Tanzania (USD340 million [4]). Furthermore, an estimated 68% of the population in 2007 live on less that USD1.25 a day [41] suggesting that their daily income was consumed entirely due to governance failures for each fever episode.

## Conclusions

We investigated the behaviour and expenditure of households during fever seeking in the Ulanga district in Tanzania in 2010 -2011 when the district, like the rest of the country, was experiencing frequent periods of ACT stock-out. The main governance issue affecting the proper delivery of health services during a fever episode observed in our case-study was non-compliant health worker behaviour at public health facilities. Among other things, this results in decreasing the cost gap between the private and public sector. Had the public sector workers been fully compliant, treatment costs in the private sector would be significantly larger than those in the public sector. In addition to overcharging for medicines and services, another element of public sector worker non-compliant behaviour was ACT stock-outs, which we estimate increased the cost of care during fever treatment by 21% compared to treatment costs during a stock-in. For those seeking care during an ACT stock-out, the majority went to the private sector. Costs in the private sector were 19% higher than the public sector which would explain the higher costs for those seeking treatment during an ACT stock-out.

As the ACT stock-out rate in our case-study was low compared to the district average, we were unable to fully assess its impact. However, our results demonstrated that there are other important governance challenges in the health system that increase household expenditure during care-seeking for fever. Now that *SMS for Life* is operating at a national level and as other household costing data sources become available, we encourage a larger scale study to assess the national impact on household expenditures of an ACT stock-out. Furthermore, an investigation into the long term health consequences of people not accessing the first line antimalarial would be important for health services management policy. With potential ACT resistance, it is imperative that Tanzania secures a stable ACT supply with adequate buffer stock and implements the use of RDTs to provide the population with an affordable treatment against one of the major public health concerns and work towards the goal of universal coverage for malaria treatment.

## Endnote

^a^Masanja, H. et al (2005) Ifakara Health Institute, Dar es Salaam.

### Key messages

•Public health facilities in Tanzania have experienced frequent prolonged stock-out episodes of the first line antimalarial treatment (ACT) since 2009.

•Non-compliant health worker behaviour was observed for 54% of patients attending public sector facilities at District level in Tanzania.

•Non-compliant health worker behaviour increased out-of-pocket household expenditures in the public sector by a factor of twelve.

•Health system design and governance issues need to be addressed to reduce this additional cost burden to households.

## Competing interests

The authors declare that they have no competing interests.

## Authors’ contributions

DDS, IML and FT contributed equally to the conceptualisation and design of the approach. RK and FM led the work in the field. FM, MN, GA, BA and IML managed and analyzed data. IML, FT, and DDS wrote the manuscript and all authors reviewed, contributed to, and approved the final manuscript.

## Pre-publication history

The pre-publication history for this paper can be accessed here:

http://www.biomedcentral.com/1472-6963/13/444/prepub
